# Scaling trends and performance evaluation of 2-dimensional polarity-controllable FETs

**DOI:** 10.1038/srep45556

**Published:** 2017-03-30

**Authors:** Giovanni V. Resta, Tarun Agarwal, Dennis Lin, Iuliana P. Radu, Francky Catthoor, Pierre-Emmanuel Gaillardon, Giovanni De Micheli

**Affiliations:** 1 Integrated System Laboratory (LSI), School of Engineering, École Polytechnique Fédérale de Lausanne (EPFL), CH-1015 Lausanne, Switzerland; 2 IMEC, Kapeldreef 75, B-3001 Leuven, Belgium; 3 KU Leuven, Celestijnenlaan 200D, B-3001, Leuven, Belgium; 4 Laboratory of NanoIntegrated Systems (LNIS), Department of Electrical and Computer Engineering, University of Utah, Salt-Lake City, Utah 84112, USA

## Abstract

Two-dimensional semiconducting materials of the transition-metal-dichalcogenide family, such as MoS_2_ and WSe_2_, have been intensively investigated in the past few years, and are considered as viable candidates for next-generation electronic devices. In this paper, for the first time, we study scaling trends and evaluate the performances of polarity-controllable devices realized with undoped mono- and bi-layer 2D materials. Using ballistic self-consistent quantum simulations, it is shown that, with the suitable channel material, such polarity-controllable technology can scale down to 5 nm gate lengths, while showing performances comparable to the ones of unipolar, physically-doped 2D electronic devices.

Miniaturization of silicon-based CMOS devices has been the main drive of the silicon industry for nearly half a century, and has allowed an exponential increase in computing power, as embodied by Moore’s law. With physical gate lengths slowly approaching 10 nm, the limits of current silicon technology are becoming increasingly difficult to overcome, and new semiconductor materials and novel device concepts have been studied, that could ultimately outperform silicon^1^,^2^. Among the materials that have been studied as a semiconducting channel for charge-based devices, 2-dimensional (2D) materials of the transition-metal-dichalcogenide (TMDCs) family^3^ are one of the most exciting and promising opportunities, thanks to their electrical and physical properties^4^,^5^. The presence of a sizeable bandgap (1~2 eV) makes TMDCs materials appealing for electronics applications, as it allows us to realize devices with low leakage floor and high ON/OFF current ratios^6^,^7^,^8^,^9^,^10^. Amongst the other remarkable features of TMDCs, their layered structure provides 2D films of controllable uniform thickness with dangling-bonds free interfaces. Moreover, their extreme thinness and low in-plane dielectric constant alleviate short-channel effects (SCE) and drain-induced-barrier-lowering (DIBL)^11^,^12^, which are detrimental to device performances. The high effective mass of charge carriers (especially with respect to III-V materials) helps reducing direct source-to-drain tunneling at ultra-scaled dimensions^13^,^14^, providing a better control of the device OFF-state by the gate terminals. Furthermore, 2-dimensional materials are attractive for monolithic integration on top of CMOS or multi-stacking of TMDCs layers^15^, thanks to the low thermal budget needed in the fabrication process.

The most studied material of the TMDCs family, MoS_2_, has proven to be a viable solution for the realization of *n-*MOS transistors^6^,^7^, and ultra-scaled *n-*type devices have been recently demonstrated^16^,^17^. Short channel MoS_2_ *p-*type FETs fabricated with doped silicon contacts^18^ have also been reported, however, MoS_2_ has not experimentally shown any ambipolar behaviour, which is essential for the realization of polarity-controllable devices. Reports of ambipolar contacts to MoS_2_ are in fact limited to devices realized on thick flakes on a PMMA substrate^19^ or devices gated with ionic liquids^20^. So far, the most promising material for the realization of both *n-* and *p-*type devices is arguably tungsten diselenide (WSe_2_), for which high carrier mobility^21^, ambipolar behavior^22^ and CMOS devices have been reported experimentally^8^,^9^. The ambipolar behavior of WSe_2_ has recently been exploited to realize polarity-controllable devices, based on undoped Schottky-barrier (SB) double-independent-gate (DIG) FETs^23^, as shown in Fig. 1. The device, presented in Fig. 1a, was experimentally realized on a WSe_2_ flake, and buried DIG gates were used to control its polarity and ON/OFF status^23^. The need for physical doping of the devices is eliminated, and the Schottky barriers created at the source and drain contact are tuned by an additional gate, namely program gate (PG), in order to select the charge carriers that can be injected in the channel. This class of devices allows the dynamic selection of the transistor polarity by the use of the PG, acting at the contact interfaces, while the control gate (CG), placed in the central region of the channel, controls the ON/OFF status of the device, as measured in Fig. 1(b,c).

The possibility of using electrostatic doping to control the device polarity avoids any complicated doping step during the fabrication process, to the benefit of fabrication simplicity and device regularity. In fact, no separate fabrication process is needed for *n-* or *p-*type devices, as the polarity can be dynamically controlled at runtime by the PG. Moreover, the device switching properties become more expressive, as each device is now acting as a comparison-driven switch and will allow the realization of compact logic gates, thus improving the computational density in 2D-flatronics^23^,^24^. However, to date, scaling opportunities with 2D materials have been theoretically explored only in unipolar, physically-doped devices, with Ohmic contacts^11^,^12^,^13^,^14^,^25^. This has been done disregarding the great difficulties that the accurate and controlled doping of the material brings to the fabrication process, i.e., doping is already one of the major sources of variability in silicon CMOS devices^26^, and that achieving Ohmic contact to 2D materials has, so far, proven to be a challenging task. Here we study, for the first time, scaling opportunities for polarity-controllable devices based on 2D materials of the TMDCs family. To estimate the electrical characteristics of such ultra-scaled devices, we use ballistic self-consistent quantum simulations in the non-equilibrium Green’s function (NEGF) formalism, as described in Methods. We first explore scaling for devices based on WSe_2_, the most promising material for which experimental results, presented in Fig. 1, are available^23^, and then focus on the selection of novel 2D semiconductor, for which experimental demonstrations are still lacking, to enhance the performances of the device. We show that such device can achieve performances that are comparable to unipolar doped devices with Ohmic contacts simulated with a similar approach, while bringing considerable simplifications to the fabrication process and bearing the promise of enhanced performances at circuit level.

## Methodology

**Figure 2:**
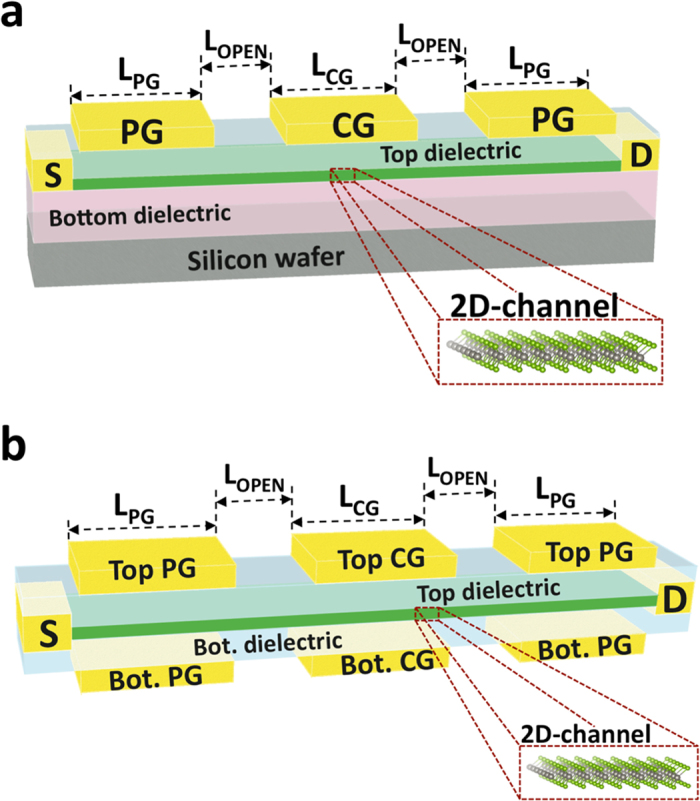
3D schematic of the simulated devices. (**a**) Topgate (TG) device structure. (**b**) Double-gate (DG) device structure. In both schematics the semiconducting 2D channel is highlighted, with its atomic structure shown in the dashed boxes.

Figure 2 shows the 3D schematic structures of the simulated devices with top-gate (TG) and double-gate (DG) geometry (Fig. 2(a,b), respectively). In the top-gate configuration, HfO_2_ (κ = 25, equivalent oxide thickness (EOT) = 0.47 nm) was used as top dielectric, while SiO_2_ (κ = 3.9, EOT = 30 nm) was considered as bottom dielectric. For the double-gate geometry HfO_2_ (κ = 25, EOT = 0.47 nm) was used for top and bottom gate dielectrics. We modeled the 2D semiconducting channel with a 2-band tight-binding (TB) Hamiltonian, created from the material properties shown in Table 1 (see also Methods).

The model was extended to bilayer 2D materials by adding an interlayer hopping parameter in the effective-mass Hamiltonian, to account for coupling between the two layers^27^. We studied the device switching properties performing self-consistent ballistic simulations, iteratively solving Poisson and Schrödinger equation (within the NEGF formalism), with an open-source quantum transport code^28^,^29^. No doping was introduced at source and drain contacts for both gate geometries and we assumed mid-gap SB contacts, to have symmetric characteristics for the two polarities. We evaluated the device performances at different gate lengths, keeping the same length for both the CG and PG gates (L_CG_ = L_PG_) and fixing the length of the ungated channel region (L_OPEN_), separating PG and CG, to L_CG_/2, as shown in Fig. 2. Thus, in the remainder of the article, we will refer to L_G_ as the length of each gated segment. The program gates are placed in close proximity to source and drain contact (an underlap of 0 nm is used in all simulations) in order to provide the most efficient modulation of the Schottky barrier. For each simulated transfer characteristic, the value of the voltage applied to the program gate (V_PG_) was fixed, thus setting the device polarity, and the switching properties as a function of the control gate voltage (V_CG_) were studied.

## Results and Discussion

**Figure 3:**
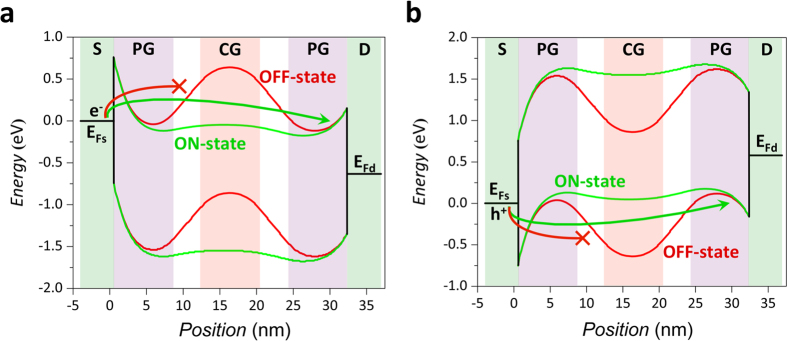
Band-diagrams of the 4 regions of operation extracted from the simulation with monolayer WSe_2_ at LG = 8 nm. (**a**) *n*-type operation, for V_PG_ = 1 V. The program gate (PG) sets the polarity of the device, by thinning the Schottky barrier (SB) for electrons (e^−^) at source and drain, while the control gate (CG) controls the ON/OFF switching of the FET. In the OFF-state (V_CG_ = 0 V), the potential barrier, created in the channel by the CG, blocks electron conduction from source to drain (red crossed line). In the ON-state, with the band diagram extracted at V_CG_ = 0.8 V, the barrier is removed and electron conduction takes place (green arrow). (**b**) *p-*type operation for V_PG_ = −1 V. In this case, the negative voltage applied to the PG enables holes (h^+^) to be injected in the channel at source (green arrow). In a similar way, as described for *n*-type operation, the potential barrier created by the CG blocks the flow of holes from source to drain (red crossed line).

The operation principle of the device is shown in Fig. 3 with the help of the band-diagrams extracted from the simulations on monolayer (1 L) WSe_2_ at L_G_ = 8 nm. The PG controls the device polarity by tuning the effective Schottky barriers height (*ϕ*_*SB*_) at source and drain (*n-*type behavior at V_PG_ = 1 V in Fig. 3a and *p-*type behavior at V_PG_ = −1 V in Fig. 3b) while the control gate (CG) determines the ON/OFF state of the FET by controlling the potential barrier in the central region of the channel. Our simulation results show that the polarity of the device can be controlled at ultra-scaled dimensions, down to 4 nm gate lengths, when direct tunneling through the CG potential barrier begins to considerably degrade the device OFF-state.

**Figure 4:**
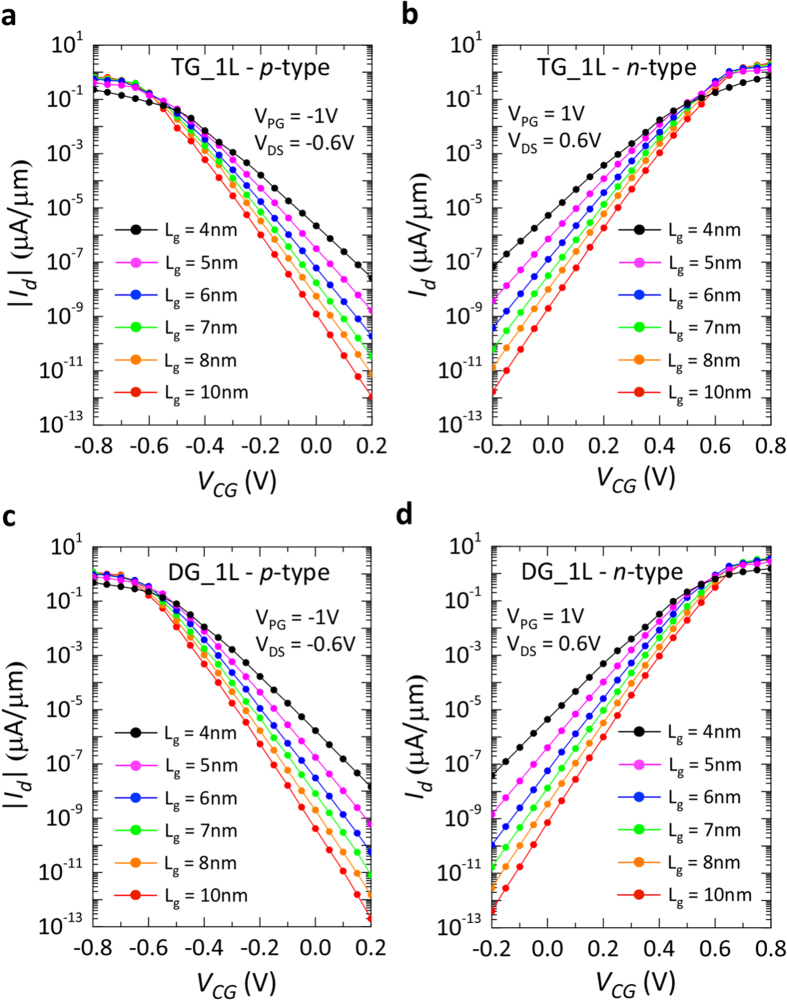
Simulated transfer characteristics for monolayer-WSe_2_ polarity-controllable FETs at different gate lengths. Monolayer-WSe_2_ was modeled with 1.5 eV bandgap and the hopping parameters of the effective mass Hamiltonian were calculated using an effective mass (m_e_) of 0.33 m_0_ for electrons and of 0.45 m_0_ for holes. The Schottky barrier height (*ϕ*_*SB*_) was set to 0.75 eV for both charge carriers, simulating a mid-gap Schottky contact. (**a,b**) Transfer characteristics of *p-* and *n-*type FET with top-gated geometry. The gate length is varied from 10 nm down to 4 nm. (**c,d**) Transfer characteristics of *p-* and *n-*type FET with double-gated geometry. The gate length is varied from 10 nm down to 4 nm.

Figure 4 shows the simulated *p-* and *n-*type transfer characteristics for 1L-WSe_2_ channel, with TG (Fig. 4(a,b)) and DG (Fig. 4(c,d)) geometry. The gate length is varied to show the impact of scaling on the device characteristics. It is found that 1L-WSe_2_ provides excellent control of the device OFF-state, thanks to the high bandgap (~1.5 eV)^30^, but also severely limits the ON-current of the device due to the high Schottky barrier (*ϕ*_*SB*_ = 0.75 eV) present at source, where carriers are injected in the channel. The modulation induced by the PG at ±1 V is enough to show conduction of charge carriers, but the ON-currents only reach values of a few μA/μm for DG geometry.

**Figure 5:**
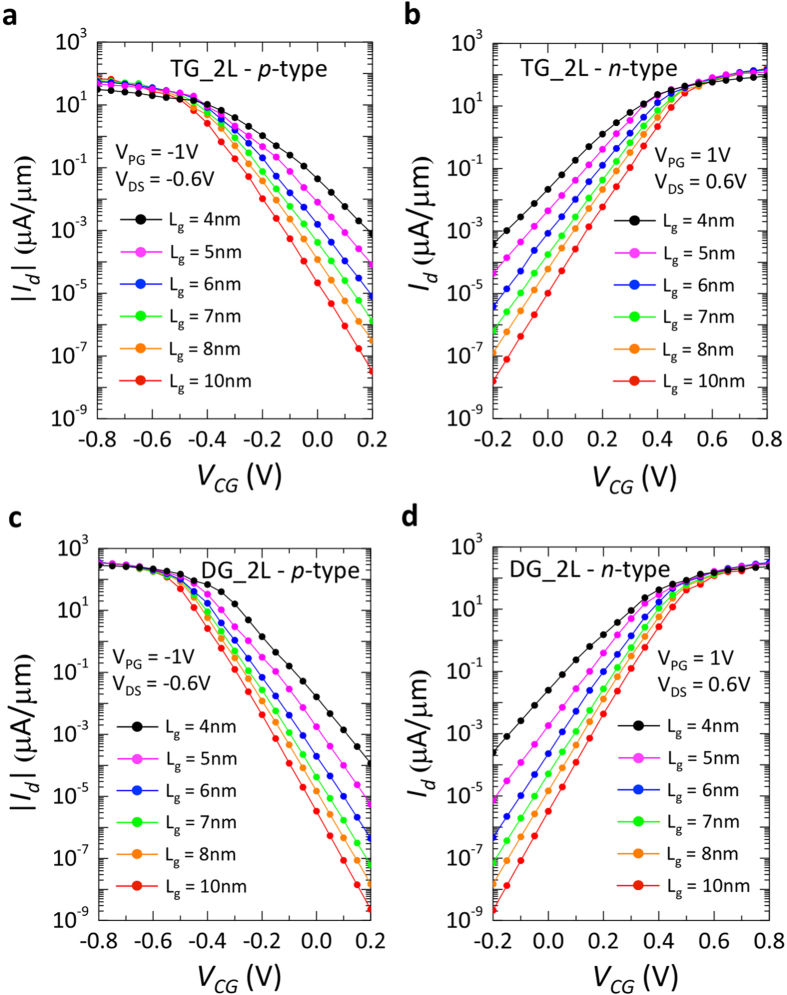
Simulated transfer characteristics for bilayer-WSe_2_ polarity-controllable FETs. 2L-WSe_2_ was modeled with 1.1 eV bandgap and the hopping parameters of the effective mass Hamiltonian were calculated using an effective mass (m_e_) of 0.33 m_0_ for electrons and of 0.45 m_0_ for holes. An interlayer hopping parameter was added to the Hamiltonian to account for interlayer coupling. The Schottky-barrier height (*ϕ_SB_*) was set to 0.55 eV for both charge carriers, simulating a mid-gap Schottky contact. (**a,b**) Transfer characteristics of *p-* and *n-*type FET with top-gated geometry. The gate length is varied from 10 nm down to 4 nm. (**c,d**) Transfer characteristics of *p-* and *n-*type FET with double-gated geometry. The gate length is varied from 10 nm down to 4 nm.

Therefore, to increase the ON current of the devices, bilayer (2 L) WSe_2_ was studied as a channel material. In its bilayer form WSe_2_ shows a reduced bandgap of ~1.1 eV^31^, which together with the increased mobile charge density, provided by the additional layer, is predicted to improve the device ON-state. Figure 5 shows the simulated transfer characteristics of 2L-WSe_2_ FETs for both polarities and gate geometries, at different gate lengths.

**Figure 6:**
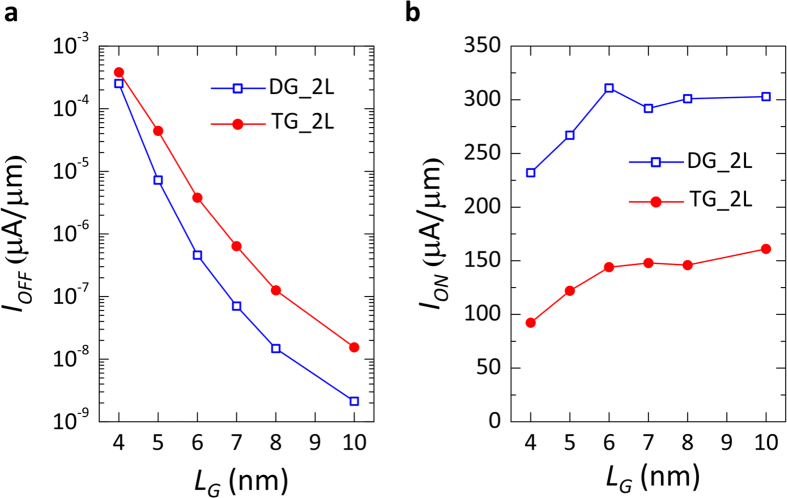
Benefits of double-gate geometry in 2L-WSe_2_ *n-*type FETs. (**a**) Comparison between I_OFF_ extracted at V_CG_ = −0.2 V and V_PG_ = 1 V for top- and double-gate devices, at different gate lengths. (**b**) Comparison of devices I_ON_ extracted at V_CG_ = 0.8 V and V_PG_ = 1 V at different gate lengths.

As a result of the decrease in Schottky-barrier height at the contact interface (*ϕ*_*SB*_ = 0.55 eV), the ON-currents are increased by 2 orders of magnitude. With the lowering of the semiconducting bandgap, the potential barrier created by the CG in the OFF-state of the device is also decreased, deteriorating the device OFF-current. The I_OFF_ is increased by almost 3 orders of magnitude. Nevertheless, the transfer characteristics presented in Fig. 5, show that even at the shortest gate length simulated (L_G_ = 4 nm), I_OFF_ is still on the range of 10^−4^ μA/μm, providing I_ON_/I_OFF_ > 10^6^. The use of a DG geometry benefits the electrostatic control of the gates over the channel, and eliminates the charge screening effect between the layers that occurs in the TG structure. The improvement in the device electrostatics, given by the DG configuration, is shown in Fig. 6 where the I_OFF_ and I_ON_ (Fig. 6(a,b) respectively), extracted from the transfer characteristics of *n-*type devices with TG and DG structures, are compared. It is found that, until L_G_ = 5 nm, the OFF-current in the DG configuration is consistently 1 order of magnitude lower with respect to the TG geometry, while the ON-current shows an average 2× improvement. For L_G_ = 4 nm, the potential barrier created by the CG starts to become thin enough to have tunneling effects, deteriorating the OFF-state of the device and thus lowering the positive impact of the double-gate. Similar results can be found for the *p-*type characteristics simulated on the same device.

**Figure 7:**
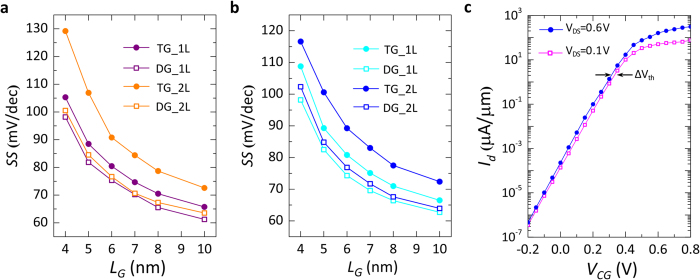
Evaluation of sub-threshold slope and DIBL. (**a**) Sub-threshold slope extracted from the transfer characteristics of *p-*type devices, for both mono- and bi-layer WSe_2_. (**b**) Sub-threshold slope for *n-*type devices. It is shown that for both polarities, the use of the double-gate geometry benefits the sub-threshold behavior by reducing the SS. (**c**) DIBL evaluation at L_G_ = 6 nm for DG *n-*type device. A V_th_ shift (ΔV_th_) of approximately 25 mV is present, leading to a DIBL of ~50 mV/V.

Further analysis is presented in Fig. 7, where the effect of scaling on the sub-threshold slope (SS) and on the drain-induced barrier lowering (DIBL) is analysed. The SS is evaluated as the average slope of the transfer characteristics in the sub-threshold regime (from −0.2 to 0.2 V_CG_) for both *p-* and *n-*type operation mode (Fig. 7(a,b) respectively). For both polarities, it is shown that the SS greatly benefits from the double-gate geometry, which is able to mitigate the detrimental effect of increased channel thickness for the bilayer device. The DIBL is calculated as the variation of threshold voltage (V_th_) of the device divided by the variation of applied V_DS_ (DIBL = ΔV_th_/ΔV_DS_) and is expressed in mV/V. A threshold voltage shift of ~25 mV can be estimated as the lateral shift, at the end of the subthreshold regime, between the transfer characteristic simulated at V_DS_ = 0.1 V and 0.6 V (see Fig. 7c). Thus we computed a DIBL of 50 mV/V for L_G_ = 6 nm, showing excellent immunity to DIBL effects. The observed immunity to DIBL is an added benefit of the SB polarity-controllable FETs, as the drain voltage drop in the channel is concentrated at the Schottky junction at drain. The change in V_DS_ does not affect the height of the potential barrier created by the CG, which is ultimately responsible for the lowering of the threshold voltage of the device.

**Figure 8:**
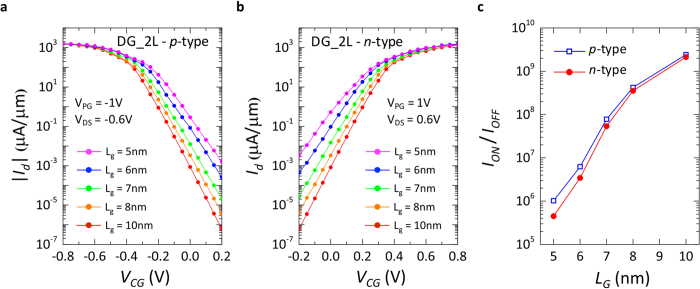
Analysis of performances for double-gate polarity-controllable device with 2L-MX_2_ material. The improved MX_2_ material was modeled with 0.8 eV bandgap, which results in a Schottky-barrier height (*ϕ*_*SB*_) of 0.4 eV. The effective masses used were m_e_ = 0.3 and m_h_ = 0.4. (**a**) Transfer characteristics for *p-*type behavior, with L_G_ varied from 10 nm down to 5 nm. (**b**) Transfer characteristics for *n-*type behavior, with L_G_ varied from 10 nm down to 5 nm. (**c**) I_ON_/I_OFF_ for both *p-* and *n-*type behavior. In both cases, I_ON_/I_OFF_ > 10^5^ is shown down to L_G_ = 5 nm.

These analyses showed that the double-gate geometry provides the best electrostatic control and enhances the performances of the device by lowering the I_OFF_, while improving the I_ON_ and SS. Nevertheless, the I_ON_ reachable with 2L-WSe_2_, in both *n-* and *p-*type operation mode, are still too low to provide a successful scaling path with this material. The Schottky barriers at source and drain (*ϕ*_*SB*_ = 0.55 eV) are too high to have efficient tunneling at the contact interface. However, theoretical calculations^32^,^33^,^34^ have shown that in the family of 2D-TMDCs, several materials, such as ZrS_2_, HfS_2_, HfSe_2_, etc., have a lower semiconducting band-gap (0.7–0.9 eV) and could prove to be well suited for application in SB-DIG FETs. For many of these materials experimental evidences are still absent or very limited^35^,^36^,^37^,^38^,^39^, and even in the theoretical ab-initio calculations there are discrepancies in the computed material properties^32^,^33^,^34^ (with great variations especially in the value of the semiconducting band-gap, depending of the functional used in ab-initio simulations). Based on these theoretical analyses, we modeled a 2D-material, according to the properties presented in Table 1, and studied its potential application as a semiconducting channel in polarity-controllable FETs. We considered a 2L-MX_2_ material with an increased lattice constant, a lower bandgap and similar effective masses with respect to WSe_2_ (as it is predicted for ZrS_2_, HfS_2_, HfSe_2_). Figure 8(a,b) shows the transfer characteristics at different L_G_ for a DG geometry for both *p-* and *n-*type polarities, while the device performances in terms of I_ON_/I_OFF_ ratios are presented in Fig. 8c. The lower Schottky-barrier height at source and drain (*ϕ*_*SB*_ = 0.4 eV) allow for a greater number of carriers to be injected in the channel, increasing the I_ON_ to ~1.5 mA/μm, while keeping I_OFF_ well below 10^−2^ μA/μm down to L_G_ = 5 nm. The lower I_ON_/I_OFF_ ratios for *n-*type behaviour shown in Fig. 8c, are caused by the lower effective mass of electrons, which increases the transmission probability of carriers over the potential barrier created by the CG, thus increasing the I_OFF_.

## Conclusions

We evaluated scaling trends and device performances for 2D polarity-controllable FETs using self-consistent ballistic quantum-transport simulations. The device concept presents the great advantage of using only a single 2D channel material for both device polarities and does not require complex doping techniques. We showed the feasibility of controllable-polarity behaviour at the nanoscale level thanks to the additional program gate introduced in the device geometry. We first simulated the performances of mono- and bi-layer WSe_2_, as a channel material, and found that the high semiconducting band-gap (~1.5 eV and 1.1 eV respectively) prevents achieving high ON-currents. Thus we studied the benefits of bilayer-MX_2_ materials, such as ZrSe_2_, HfS_2_, or HfSe_2_, for which ab-initio simulations have shown the presence of a lower semiconducting bandgap (0.7–0.9 eV). Due to the lack of experimental characterization and the disagreement between different ab-initio simulations, we modeled a bilayer-MX_2_ with electrical properties (effective masses and bandgap) within the values reported in literature^32^,^33^,^34^. For the simulated MX_2_ material, we showed I_ON_ > 10^3^ μA/μm and I_ON_/I_OFF_ > 10^5^ down to L_G_ = 5 nm for both *p-* and *n-*type polarities. These performances are comparable with the ones predicted, using ballistic self-consistent transport simulations^7^,^8^, for conventional doped devices based on 2D-TMDCs, and thus show a feasible scaling path for 2-dimensional polarity-controllable devices for beyond-CMOS flatronics.

## Methods

### Material properties and Device simulations

To perform quantum simulations within NEGF formalism, we use a 2-band tight-binding Hamiltonian to model the conduction and valence band of a chosen material^40^. We calculated the hopping parameter t_hop_, to be used by the NanoTCAD ViDES^28^,^29^ in the NEGF simulations, as^40^:


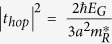


where *a* is the lattice constant, *E*_*G*_ is the energy band-gap, 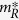 is the reduced effective mass and *ħ* is the reduced Plank constant. Here, the material parameters such as lattice constant, effective masses and band-gaps are taken from literature^30^,^31^,^32^,^33^,^34^ and reported in Table 1. This approach has been widely used to project performance of nanoscale transistors based on Si, III-V^41^ and now 2D materials^11^,^12^,^13^,^14^. Further, to model Schottky contacts, we extend our Hamiltonian at the contacts for the zero-bandgap metal and applied Dirichlet boundary conditions. This model provides a good trade-off between accuracy and computational time which is crucial in advanced device design with exotic materials for future technology nodes.

## Additional Information

**How to cite this article:** Resta, G. V. *et al*. Scaling trends and performance evaluation of 2-dimensional polarity-controllable FETs. *Sci. Rep.* **7**, 45556; doi: 10.1038/srep45556 (2017).

**Publisher's note:** Springer Nature remains neutral with regard to jurisdictional claims in published maps and institutional affiliations.

## Figures and Tables

**Figure 1:**
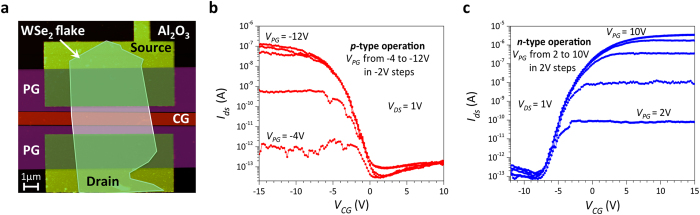
Experimental demonstration of polarity-controllable behavior in WSe_2_. (**a**) AFM topography image of the experimental device, recolored to highlight the device structure. Both the PG and CG were realized as bottom-gates. The thickness of the flake was 7.5 nm. (**b**) *p-*type transfer characteristics measured sweeping the voltage applied to the control gate (V_CG_) at different negative V_PG_ voltages. (**c**) *n-*type transfer curves measured on the same device with positive voltages applied to the PG. The experimental device had 1.5 μm channel length and 5.5 μm channel width.

**Table 1:** Material properties used to construct the effective mass Hamiltonian.

	1L WSe_2_	2L WSe_2_	2L MX_2_
E__G__(eV)	1.5	1.1	0.8
ϕ_*SB*_ (eV)	0.75	0.55	0.4
m_e_	0.33	0.33	0.3
m_h_	0.45	0.45	0.4

a is the lattice constant, E_G_ the bandgap, *ϕ*_*SB*_ the Schottky-barrier height at source and drain and m_e_, m_h_ are the effective masses.
